# A revisited phylogeography of *Nautilus pompilius*

**DOI:** 10.1002/ece3.2248

**Published:** 2016-06-21

**Authors:** Lauren E. Vandepas, Frederick D. Dooley, Gregory J. Barord, Billie J. Swalla, Peter D. Ward

**Affiliations:** ^1^ Friday Harbor Laboratories Biology Department University of Washington Seattle Washington; ^2^ Department of Biology Graduate Center City University of New York New York City New York 10016; ^3^ Department of Biology Brooklyn College City University of New York Brooklyn New York 11210

**Keywords:** Indo‐Pacific, mitochondrial DNA, *Nautilus pompilius*, phenotypic plasticity, phylogeography

## Abstract

The cephalopod genus *Nautilus* is considered a “living fossil” with a contested number of extant and extinct species, and a benthic lifestyle that limits movement of animals between isolated seamounts and landmasses in the Indo‐Pacific. Nautiluses are fished for their shells, most heavily in the Philippines, and these fisheries have little monitoring or regulation. Here, we evaluate the hypothesis that multiple species of *Nautilus* (e.g., *N. belauensis*,* N. repertus* and *N. stenomphalus*) are in fact one species with a diverse phenotypic and geologic range. Using mitochondrial markers, we show that nautiluses from the Philippines, eastern Australia (Great Barrier Reef), Vanuatu, American Samoa, and Fiji fall into distinct geographical clades. For phylogenetic analysis of species complexes across the range of nautilus, we included sequences of *Nautilus pompilius* and other *Nautilus* species from GenBank from localities sampled in this study and others. We found that specimens from Western Australia cluster with samples from the Philippines, suggesting that interbreeding may be occurring between those locations, or that there is limited genetic drift due to large effective population sizes. Intriguingly, our data also show that nautilus identified in other studies as *N. belauensis*,* N. stenomphalus*, or *N. repertus* are likely *N. pompilius* displaying a diversity of morphological characters, suggesting that there is significant phenotypic plasticity within *N. pompilius*.

## Introduction

The genus *Nautilus* (Mollusca, Cephalopoda) belongs to subclass Nautiloidea that has an extensive fossil record dating back to the Devonian (Teichert and Matsumoto 1987; Kröger et al. 2011). Here, we will refer to *Nautilus* as the genus and nautilus when discussing the animal itself. Because members of the extant *Nautilus* genus have been hypothesized to have evolved in their current form between 7 and 10 mya (Ward 1984) or possibly much earlier, approximately 40 mya (Teichert and Matsumoto 1987; Woodruff et al. 1987), and modern nautiluses appear to be very similar to some of their Mesozoic ancestors (Ward and Saunders 1997), these animals have been described as “living fossils” (Sinclair et al. 2011). The family Nautilidae (*Nautilus* Linnaeus 1758) has a disputed number of extant species ranging from two to nearly a dozen (Saunders and Landman 1987; Wray et al. 1995). The genus *Allonautilus* Ward and Saunders 1997 has one accepted species, *Allonautilus scrobiculatus*, and a second possible species, *Allonautilus perforates* (Ward and Saunders 1997). All known populations are thought to live at depths of about 100–600 m along fore‐reef slopes, with a wide distribution of the Indo‐Pacific (tropical north and south regions of the western Pacific and Indian Ocean). Extant nautilids are limited in their ability to disperse: they are obligately nektobenthic, do not swim far off the sea floor, and have rarely been observed in mid‐water (Ward et al. 1984; Dunstan et al. 2011a; P. D. Ward, pers. observ.). Nautilus have a maximum depth limit caused by fatal shell implosion of between 700 and 800 m (Ward et al. 1980; Saunders and Ward 1987) and a minimum water depth constrained by water temperatures in excess of 28°C. As most shallow waters across the range of the nautiluses' habitats is warmer than this, these high surface water temperatures and the presence of visual predators make dispersal in surface and near surface waters rare (O'Dor et al. 1993; Carlson 2010; Williams et al. 2012).

There have been an increasing number of investigations into the morphological and genetic diversity of the genus *Nautilus* (Saunders 1981; Saunders and Landman 1987; Saunders et al. 1989; Ward and Saunders 1997; Sinclair et al. 2007, 2011; Bonacum et al. 2011; Dunstan et al. 2011b; Williams et al. 2015a, 2015b). Original species descriptions of *Nautilus* utilized few discrete characters, and many of the morphological characteristics delimiting the species may be difficult to quantify or have values that overlap broadly between multiple species (e. g. size, Table S1). Confounding this is the potential for the variation of characters like shell color and size within populations. Species of *Nautilus* for which we have sequencing data available are described in Tables S1 and S2, although the validity of several of these species (*Nautilus repertus*,* Nautilus stenomphalus*, and *Nautilus belauensis*) has been questioned (reviewed in Saunders 1987).

**Figure 1:**
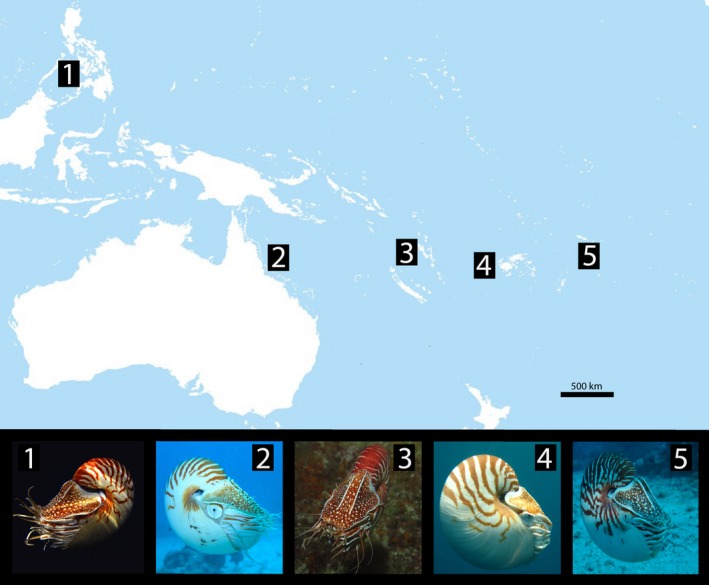
Map of the Indo‐Pacific showing sampling locations of *Nautilus pompilius* for this study and photographs of representative animals from each location: (1) Panglao, Philippines; (2) Great Barrier Reef, Australia; (3) Vanuatu; (4) Fiji; (5) American Samoa. There appears to be interesting phenotypic plasticity displayed between different populations of *Nautilus pompilius* in traits such as size, shell coloration, and hood morphology.

Although genetic work shows that different populations of *Nautilus pompilius* around disparate island groups and land masses can form clades based on location (Sinclair et al. 2007, 2011; Bonacum et al. 2011; Williams et al. 2012, 2015b), the samples between locations were few and the power of these observations may be low. A larger question that remains unresolved is whether genetic studies support several named species falling into their own distinct clades. Few studies to date have examined sequence data from multiple species of *Nautilus* (Wray et al. 1995; Bonacum et al. 2011), and the status of three taxonomic species (*N. repertus*,* N. stenomphalus*, and *N. belauensis*) remained unresolved. Previous studies have not examined nautilus samples across their entire range, nor included robust genetic analyses examining the validity of these contested species. In this study, we sought to assess whether low morphological diversity within the genus *Nautilus* reflects a low number of genetic species or whether there may be cryptic diversity within extant nautilids that is not obvious with morphology alone (Fig. 1, Table S1).

Here, we report the genetic analysis of mitochondrial genes cytochrome *c* oxidase I (COI) and 16S rDNA, commonly utilized genetic tools for the phylogeographical studies of marine invertebrates, including cephalopods (Anderson 2000; Anderson et al. 2007; Dai et al. 2012; Sales et al. 2013a) from individuals across the known locations of *Nautilus* populations (Philippines, Fiji, American Samoa, Vanuatu, and eastern Australia – Great Barrier Reef). We chose COI and 16S because of their variability and success in past studies, and to align with sequences generated for this study with previous nautilus studies (Bonacum et al. 2011; Williams et al. 2012). We neglect nuclear genes (e.g., 28S or histone 3) because sequencing efforts have been limited in nautilus, precluding comparative analysis with past studies, and have been shown to be relatively uninformative for phylogenetic studies within this genus (Wray et al. 1995). We use several analyses to understand the genetic distance between populations in hopes of shedding light on the possibility of multiple distinct populations or one highly plastic population with gene flow that is low but not significant enough to promote speciation.

## Materials and Methods

### Sample sites

Sample sites included broad geographical ranges in the Indo‐Pacific at locations with known nautilus populations (Philippines, Australia, Vanuatu American Samoa, and Fiji). In the Philippines, samples were collected in the Bohol Sea (9°35′18.87″N, 123°43′44.54″E) off the coast of Panglao. In Australia, we collected along a transect of the Great Barrier Reef from Cairns to Lizard Island (16°37′28.91″S, 145°53′07.35″E). In Fiji, we samples in Beqa Harbor near Pacific Harbor (18°19′40.24″S, 178°06′30.86″E). We sampled on Taena Bank near the harbor of Pago Pago, American Samoa (14°19′19.57″S, 170°38′57.78″W) and in Mele Bay, Port Vila, Vanuatu (17°44′21.3″S, 168°15′56.7″E).

### Sample collection

Tissue sampling was conducted alongside the deployment of Baited Remote Underwater Video Systems (BRUVS; Barord et al. 2014). Traps consisted of weighted cages approximately 2 m in length and 1 m in diameter with a double entry. Traps were baited with canned tuna and raw chicken. Traps were deployed between 200 and 400 m depth. Captured nautiluses were placed into chilled seawater while on the surface and returned to their location of capture upon the completion of examination. Individuals were photographed, sexed, and weighed, and measurements of shell length and width were taken. Each individual was x‐rayed, and a tentacle snip or small pieces of hood tissue were collected for DNA extraction. Tissue samples were stored in 95% ethanol and returned to the University of Washington in Seattle, WA, USA, for processing.

### DNA extraction, PCR, and sequencing

Genomic DNA was extracted using DNeasy Blood and Tissue Kit (Qiagen, Valencia, CA). Primers for *cytochrome oxidase I* were taken from Meyer (2003): dgLCO‐1490 GGTCAACAAATCATAAAGAYATYGG (forward), dgHCO‐2198 TAAACTTCAGGGTGACCAAARAAYCA (reverse). COI amplifications were carried out in 25‐*μ*L reactions containing 12.5 *μ*L of PrimeSTAR^®^ MAX DNA Polymerase Premix (Clontech Laboratories, Mountain View, CA), 5.5 *μ*L water, 1.0 *μ*g each primer, and 25–50 ng total DNA. Cycling conditions for CO1 are 35 cycles of 10 sec denaturing at 98°C, 15 sec annealing at 48°C, and 10 sec elongation at 72°C. Primers for *16S* were described previously (Sales et al. 2013b) L1987 GCCTCGCCTGTTTACCAAAAAC (forward), H2609 CGGTCTGAACTCAGATCACGT (reverse). *16S* amplifications were carried out in 25‐*μ*L reactions containing 2.5 *μ*L 10× PCR buffer (100 mmol/L Tris–HCl pH 9, 500 mmol/L KCl, 15 mmol/L MgCl_2_, 1% Triton X‐100), 18.5 *μ*L water, 10 mmol/L dNTPs, 1.0 *μ*g each primer, 1 unit taq polymerase, and 25–50 ng total DNA. Cycling conditions for 16S were 2 m initial denaturing at 94°C, followed by 30 cycles of 30 sec denaturing 94°C, 1 m annealing at 51°C, and 2 m elongation at 72°C and 7 m at 72°C for final extension. PCR products were extracted from agarose gel using Illustra GFX PCR DNA and Gel Band Purification Kit (GE Healthcare, Pittsburgh, PA). DNA sequencing was performed using BigDye3.1 (Life Technologies, Carlsbad, CA) with a 3130 DNA Analyzer (Life Technologies) in the University of Washington Biology Department Comparative Genomics Center.

Forward and reverse sequences were generated for each sample and compared to eliminate sequencing error. The coding sequences were translated to protein sequences to verify that the reading frame was not disrupted by premature stop codons or deletions, as a further check of sequence quality and locus identity.

### Sequence alignments and phylogenetic analysis

Cytochrome oxidase I, 16S, and concatenated sequences were aligned using MUSCLE (Edgar 2004) in Geneious version 9.0.5 (Kearse et al. 2012). Optimal nucleotide substitution models were determined for each gene under the corrected Akaike information criterion (AIC_c_) in jModeltest 2.1.4 (Darriba et al. 2012). The HKY + I model was applied to 16S; HKY + G to COI; and GTR + I + G to concatenated 16S‐COI. Phylogenetic analyses using Bayesian inference in Mr. Bayes 3.1.2 (Ronquist and Huelsenbeck 2003); two independent parallel runs of four incrementally heated Metropolis‐coupled Monte Carlo Markov chains, sampling every 1000 generations with a burn‐in of 25%. Trees were visualized in FigTree v1.3.1 (Rambaut 2009). Outgroups and other *Nautilus* sequences were obtained from GenBank. Numbers of variable and informative sites were generated using PAUP* version 4.0b10 (Swofford 2002).

### Analysis of genetic diversity and structure

Divergences of concatenated 16S‐cytochrome oxidase I (COI) sequences within and between sampling location sites in our study and COI sequences from GenBank (Table S2) were estimated using average pairwise distances (p‐distances) calculated in Arlequin 3.5 (Excoffier and Lischer 2010). For population analysis for samples from our study, concatenated 16S‐COI sequences were trimmed to the same length and ambiguous base calls removed to minimize error and biases. To examine intraspecific relationships between sampling localities, a haplotype network for each mitochondrial gene and concatenated sequences were constructed based on the TCS algorithm (Clement et al. 2002) in PopART (http://popart.otago.ac.nz). Compiling all COI sequences (400 bp or longer) from GenBank, we constructed a haplotype network for samples across the entire known geographical range of *Nautilus* for which there is genetic data available.

To quantify genetic differentiation between sampling localities across the Indo‐Pacific pairwise, *F*_ST_ values were calculated Arlequin 3.5 (Excoffier and Lischer 2010). Although we could not perform a hierarchical AMOVA on samples from our study alone, which requires sampling more than one population per region or location, we utilized the entire suite of *Nautilus* spp. cytochrome oxidase subunit I (COI) sequences available on GenBank to examine genetic diversity and population connectivity of the genus. To test whether populations evolved under neutrality Tajima's *D* (Tajima 1989, 1996) was calculated with 1000 permutations in Arlequin.

## Results

### Morphological and sample information

Examples of nautilus specimens collected in each of locality are illustrated in Figure 1. Table 1 lists the shell sizes (length and width), sex, and weights of the animals collected in this study. To date, there have been few, if any, informative taxonomic features identified from soft parts of nautiluses (Saunders and Landman 1987). Because taxonomic efforts have focused on shell and hood morphology, species‐specific identification using these characters should be treated with caution, as phenotypic plasticity within and between populations is not well understood.

**Table 1:** Specimen collection location, sex, weight, shell length, shell width, and GenBank accessions for mitochondrial genes *cytochrome oxidase subunit I* (COI) and *16S* rDNA

Country, sample number	Sampling location	COI GenBank accession	16S GenBank accession	Sex	Weight (g)	Shell length (mm)	Shell width (mm)
American Samoa 1	Pago Pago, Taena Bank		KR062163				
American Samoa 3	Pago Pago, Taena Bank	KM020806	KR062164				
American Samoa 4	Pago Pago, Taena Bank	KM020805	KR108897				
American Samoa 5	Pago Pago, Taena Bank	KM020804	KR108898				
American Samoa 6	Pago Pago, Taena Bank	KM020803	KR108899				
Australia 1	Great Barrier Reef	KM020802	KR062142	M		160	79
Australia 3	Great Barrier Reef	KM020801		M		180	85
Australia 4	Great Barrier Reef	KM020800		M		155	80.5
Australia 5	Great Barrier Reef	KM020799		F		152	73.5
Australia 6	Great Barrier Reef	KM020798		M		157	81
Australia 7	Great Barrier Reef	KM020797		M		160	79
Australia 8	Great Barrier Reef	KM020796	KR062143	NA		156	78
Australia 9	Great Barrier Reef	KM020795	KR062144	M		150.5	75.5
Australia 11	Great Barrier Reef	KM020794	KR062145	M		149	77
Australia 12	Great Barrier Reef	KM020793	KR062146	M		165	79
Australia 13	Great Barrier Reef	KM020792	KR062147	M		153	78
Australia 14	Great Barrier Reef	KM020791	KR062148	M		152	71
Australia 15	Great Barrier Reef		KR062149	F		135	67.5
Australia 16	Great Barrier Reef	KM020790	KR062150	NA		143	69
Australia 17	Great Barrier Reef		KR062151	F		143	70.5
Australia 18	Great Barrier Reef	KM020789	KR062152	F		138.5	59
Australia 19	Great Barrier Reef	KM020788	KR062153	F		143	69.5
Australia 20	Great Barrier Reef	KM020787		F		144	70
Australia 22b	Great Barrier Reef	KM020786	KR062154	NA		161	81
Australia 23	Great Barrier Reef	KM020785	KR062155	M		170	82
Australia 24	Great Barrier Reef	KM020784	KR062156	M		137	78
Australia 25	Great Barrier Reef		KR062157	M		168	80
Australia 26	Great Barrier Reef		KR062158	M		156	76
Australia 27	Great Barrier Reef	KM020783	KR062159	M		182	83
Australia 28	Great Barrier Reef		KR062160	M		168	83
Australia 29	Great Barrier Reef	KM020782	KR062161	M		166	81
Australia 30	Great Barrier Reef	KM020781	KR062162	F		157	79
Fiji 1	Beqa Harbor	KM020780	KR108896		600	151.5	77
Fiji 2	Beqa Harbor	KM020779	KR062165		480	138	72.5
Fiji 3	Beqa Harbor		KR062166		600	147.5	75
Fiji 4	Beqa Harbor	KM020778	KR062167		590	141.5	72.5
Philippines 1	Bohol Sea, Panglao	KM020777	KR062176		400	128.6	67.3
Philippines 2	Bohol Sea, Panglao	KM020776	KR062177		1030	162.4	97.2
Philippines 3	Bohol Sea, Panglao	KM020775	KR062178		1050	191	92.8
Philippines 4	Bohol Sea, Panglao	KM020774	KR108880		315	126.4	70.3
Philippines 5	Bohol Sea, Panglao	KM020773	KR108881		470	146.8	82.5
Philippines 6	Bohol Sea, Panglao	KM020772	KR108882		1140	194.3	94.2
Philippines 7	Bohol Sea, Panglao	KM020771	KR108883		490	141.4	76.2
Philippines 8	Bohol Sea, Panglao	KM020770	KR108884		450	143	73.3
Philippines 9	Bohol Sea, Panglao	KM020769	KR108885		660	158	87.3
Philippines 10	Bohol Sea, Panglao	KM020768	KR108886		350	127	65.5
Philippines 11	Bohol Sea, Panglao	KM020767	KR062179		650	157.5	85.6
Philippines 12	Bohol Sea, Panglao	KM020766	KR108887		600	157.5	90
Philippines 13	Bohol Sea, Panglao	KM020765	KR108888		985	181	84
Philippines 14	Bohol Sea, Panglao	KM020764	KR062181		1125	188.7	92.1
Philippines 15	Bohol Sea, Panglao	KM020763	KR108889		1090	184.9	94.1
Philippines 16	Bohol Sea, Panglao	KM020762	KR108890		845	178	87.7
Philippines 17	Bohol Sea, Panglao	KM020761	KR108891		995	173	91
Philippines 18	Bohol Sea, Panglao	KM020760	KR108892		1140	182	90.1
Philippines 20	Bohol Sea, Panglao	KM020759	KR108893		975	186.5	89
Philippines 21	Bohol Sea, Panglao		KR108894		835	176.3	85
Philippines 22	Bohol Sea, Panglao		KR062180		1050	188.5	91.9
Philippines 24	Bohol Sea, Panglao	KM020758			1210	195.3	95.6
Philippines 27	Bohol Sea, Panglao	KM020757			1050	185.2	87.1
Philippines 28	Bohol Sea, Panglao		KR108895				
Philippines 29	Bohol Sea, Panglao	KM020756			1100	190	87.9
Vanuatu 1	Mele Bay, Port Vila	KR025872	KR062168	F	850	129.6	74.1
Vanuatu 2	Mele Bay, Port Vila	KR025876	KR062169	F	920	124.39	77.27
Vanuatu 3	Mele Bay, Port Vila	KR025873	KR062170	M	820	152	81
Vanuatu 4	Mele Bay, Port Vila	KR025874	KR062171	M	750	144	75.57
Vanuatu 5	Mele Bay, Port Vila	KR025875	KR062172	M	800	150	78.4
Vanuatu 6	Mele Bay, Port Vila		KR062173	M	860	161.46	80.39
Vanuatu 7	Mele Bay, Port Vila		KR062174	M	820	150.7	75.8
Vanuatu 8	Mele Bay, Port Vila		KR062175	F	850	154	87

### Phylogenetic relationships between *Nautilus* species and populations

**Figure 2:**
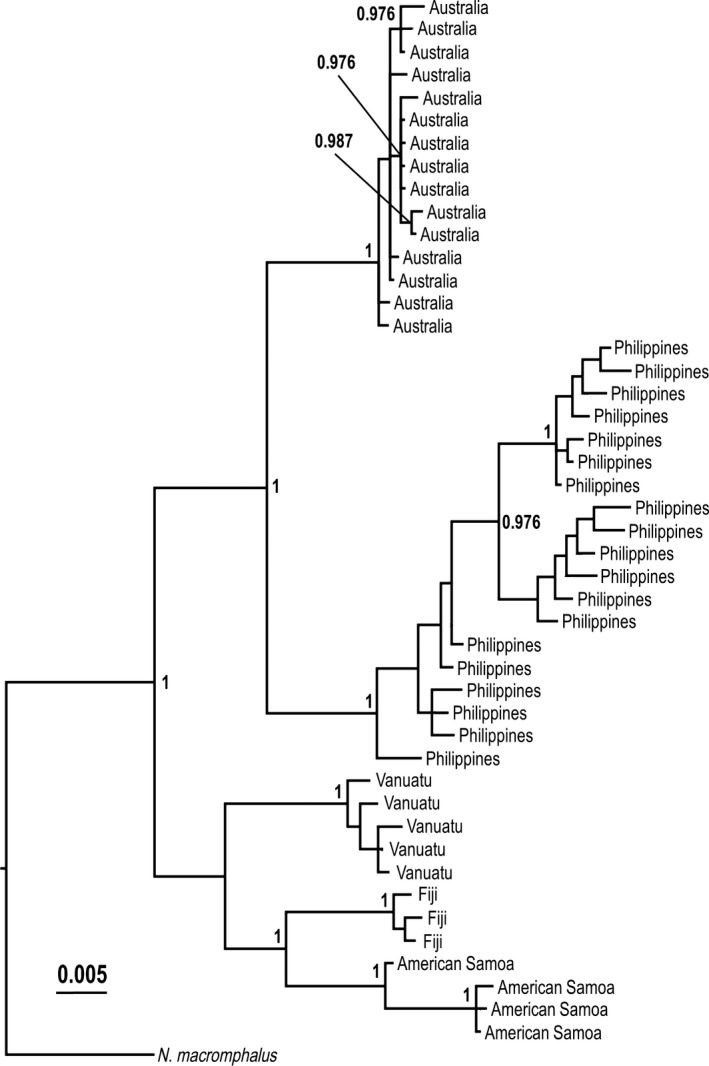
Bayesian inference tree based on 1196 bp concatenated sequences of *cytochrome oxidase subunit I* (*COI*) and *16S* rDNA sequences of *Nautilus macromphalus* (GenBank accession: NC_007980.1) and *Nautilus pompilius* from our study (See Table 1 for accessions). Samples from discrete geographical locations cluster together with high support. Posterior probabilities below 0.95 are not shown.

**Figure 3:**
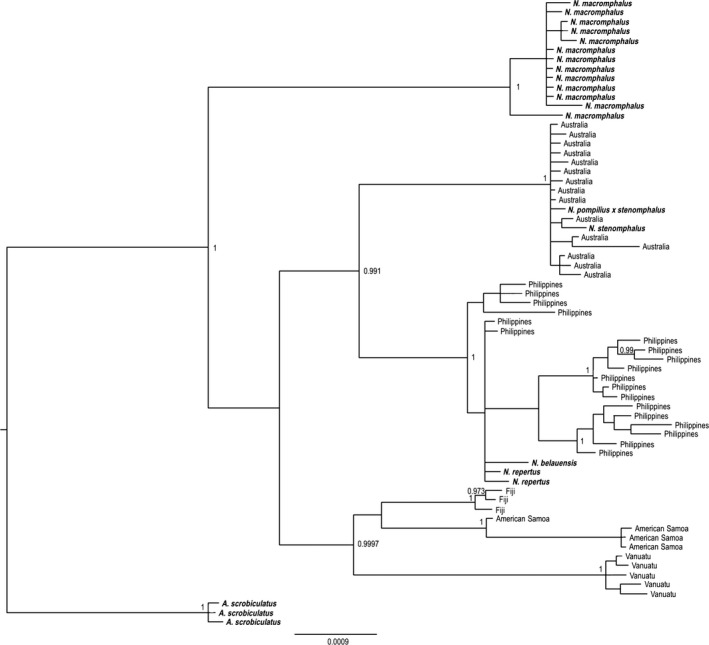
Bayesian inference tree of concatenated *COI‐16S* sequences of *Nautilus pompilius* sequences from our study, and sequences of specimens identified in other studies as separate *Nautilus* species from GenBank (in bold; accessions can be found in Table S2). Specimens identified in other studies as *Nautilus* species *N. belauensis*,* N. stenomphalus*, and *N. repertus* do not fall into discrete clades but are interspersed throughout *N. pompilius* samples. Posterior probabilities below 0.95 are not shown.

All nautiluses that we sampled were *N. pompilius*, including specimens from the GBR (Australia) (Fig. 1, Table 1). Using concatenated 16S‐COI sequences analyzed by Bayesian statistics, samples from Fiji, American Samoa, Philippines, Vanuatu, and Australia (GBR) each cluster together with high support based on geographical location, with Fiji, Vanuatu, and Samoa samples forming a sister clade to samples in the western Indo‐Pacific (Philippines and Australia) (Fig. 2). Although some individuals from the Great Barrier Reef were identified in previous studies as the species *N. stenomphalus* (Saunders 1987; Bonacum et al. 2011), our rigorous phylogenetic analyses show that sequences from these samples are indistinguishable from those of *N. pompilius* (Fig. 3). Our *16S* alignment contained 71/554 variable sites, with 44/554 being parsimony‐informative. The *COI* sequences alignment from our study contained 130/709 variable sites with 97 being parsimony‐informative. The Bayesian trees built with concatenated 16S‐COI sequences from this study and from other identified *Nautilus* species from GenBank (Fig. 3) are consistent with our conclusion that *N. repertus*,* N. belauensis*,* N. stenomphalus*, and an individual identified as a hybrid *N. stenomphalus* × *N. pompilius* do not fall within distinct clades. Rather, they are scattered within the *N. pompilius* clade.

When GenBank COI sequences from additional geographical locations were analyzed with our sequences (Figure S1), samples from Western Australia were interspersed with samples from the Philippines, and Papua New Guinea sequences clustered with samples from eastern Australia. This may indicate that the populations in these areas may not be reproductively isolated, and there is likely gene flow between the *Nautilus* populations in the Philippines (a fished population) and Western Australia (an unfished population), and between Papua New Guinea and eastern Australia. Because of the few informative sites in 16S sequences, Bayesian inference did not robustly recover geographical clades (Figure S2).

### Genetic diversity of *Nautilus* populations

When calculating the fixation index (*F*_ST_) using concatenated *16S‐COI* sequences from samples from our study alone, *F*_ST_ values between all populations (from Fiji, American Samoa, Philippines, Vanuatu, GBR – Australia) were high (0.888 for Philippines/GBR – 0.975 for American Samoa/GBR), indicating very little gene flow between these populations (Table 2, above diagonal). Using *COI* sequences from a large swath of GenBank samples (Table 2, below diagonal; Table S2), we labeled individual sampling localities (*n* = 27) as “populations,” while “groups” were geographical clusters of populations (*n* = 10), as specified in Table 3. Low *F*_ST_ values, closer to 0, indicate greater sharing of genes between populations (e.g., values between Western Australia, Indonesia, and the Philippines; Table 2, below diagonal). High *F*_ST_ values (nearer to 1) indicate isolation of a population (e.g., between American Samoa and other populations: 0.878–0.957). Significant *F*_ST_ values for *COI alone* ranged from −0.1872 (Indonesia–Philippines) to 0.97462 (Palau–Fiji). *F*_ST_ values were not significant divergence between Palau–Indonesia, Palau–Philippines, and Palau–Western Australia, indicating that there is no significant differentiation between these populations and gene flow may be occurring *F*_ST_ values between Indonesia and Philippines were also relatively low (0.1872).

**Table 2:** Population analyses for *Nautilus pompilius* samples. Above diagonal: Pairwise *F*_st_ of *Nautilus pompilius* collected for this study alone (See Table 1 for accessions), using concatenated *16S‐COI* sequences. Below diagonal: Pairwise *F*_st_ of *Nautilus pompilius COI* sequences between sampling regions (See Table S2 for accessions)

	Eastern Australia	Philippines	Vanuatu	Fiji	American Samoa	Western Australia	Indonesia	Papua New Guinea	Palau	New Caledonia
Tajima's *D* for 16S/COI	−1.43028	−1.10372	0.08298	0.00000	−0.61237					
Eastern Australia		0.88762*	0.95909*	0.96959*	0.97504*					
Philippines	0.79171*		0.89254*	0.90582*	0.91071*					
Vanuatu	0.86653*	0.90265*		0.90471*	0.91695*					
Fiji	0.8709*	0.91197*	0.93848*		0.97114*					
American Samoa	0.87821*	0.90281*	0.92109*	0.94792*						
Western Australia	0.83294*	0.20906*	0.92827*	0.93688*	0.93231*					
Indonesia	0.81541*	0.90265*	0.95373*	0.96388*	0.95748*	0.04848*				
Papua New Guinea	0.30838*	0.82014*	0.95373*	0.97605*	0.90875*	0.93231*	0.88836*			
Palau	0.79726*	0.1175	0.95264*	0.97462*	0.9547*	−0.04844	−0.03771	0.83611*		
New Caledonia	0.86982*	0.92806*	0.96578*	0.97605*	0.97313*	0.93716*	0.96388*	0.87867*	0.97019*	

Significant values (*P* < 0.05) are marked with an “*”.

**Table 3:** *Nautilus pompilius* genetic diversity analyses using *COI* sequences from this study and sequences from GenBank

Geographical group (bold)/Population	No. samples	Tajima's *D*	Mean *H*_E_	GenBank accessions
**Eastern Australia**		**0.843**		
Bougainville Reef	2	0.000	N/A	EF128187‐88
Carter Reef	6	0.939	0.506	GQ280195‐200
Osprey Reef	39	−0.692	0.162	EF128184,189,197‐210,212‐215; JQ862308‐21
Shark Reef	19	0.417	0.351	EF128195‐ 96,211
N. Great Barrier Reef	26	0.842	0.345	EF128174‐183; JN227635; JQ862293‐307
Cairns‐Lizard Isl.	22	−1.404	0.154	KM020781‐802
**Western Australia**		−**1.913** *		
Scott Reef	62	−1.328	0.121	GQ387444‐81; JQ890081‐90; JQ862322‐33
Ashmore Reef	10	−0.470	0.314	JN227639‐48
Clerke Reef	10	−0.856	0.283	JN227649‐58
Imperieuse Reef	9	−1.205	0.272	JN227659‐87
Rowley Shoals	2	0.000	1.0	
**New Caledonia**		−**1.186** *		
Ile des Pins	13	−0.909	0.218	GQ280227‐39
Noumea	10	−1.573	0.222	GQ280217‐26
**Fiji**		−**0.817**		
Suva	2	0.000	N/A	GQ280215‐16
Beqa Harbor	3	0.000	0.667	KM020778‐80
**Indonesia**		−**1.202**		
Ambon Straight	2	0.000	N/A	GQ280190‐91
Unspecified	28	−1.187	0.173	KC539898‐925
**Papua New Guinea**		−**1.666** *		
Little Ndrova Isl.	8	−0.168	0.371	GQ280206‐13
Lorengau	2	0.000	1.0	GQ280203‐04
Port Moresby	2	0.000	1.0	GQ280201‐02
Komuli Isl.	1	0.000	N/A	GQ280205
**Vanuatu**	10	−**1.146**	0.256	GQ280240‐49
**Philippines**		−**0.802**		
Bohol Sea	22	−0.569	0.231	KM020756‐77
Panglao	1	0.000	N/A	GQ280192
Balayan Bay	2	0.000	1.0	GQ280193‐94
**Palau**	4	**0.168**	0.556	GQ280187
**American Samoa**	5	−**1.094**	0.40	KM020803‐06, GQ280214

*H*_E_, expected heterozygosity.

Significant values (*P* < 0.05) are marked with an “*”.

To determine whether or not populations are geographically structured across the geographical range of *N. pompilius*, hierarchical analyses of molecular variance (AMOVAs) were performed based on pairwise differences with populations (1) treated as a single group to determine the amount of variation partitioned among and within locations, and (2) grouped into geographical clades. Hierarchical AMOVAs attributed 85.52% of the overall variation to variation across the geographical range of *N. pompilius* and only 14.48% to variation within populations (Table S3).

Tajima's *D* tests departures from population equilibrium, comparing the mean number of pairwise differences and the number of segregating sites in a sample, with positive selection expected to yield a negative Tajima's *D* value in a population with recent population expansion or purifying selection (Tajima 1989, 1996). Tajima's *D* values were significant in Western Australia (−1.193), New Caledonia (−1.186), and Papua New Guinea (−1.666) (Table 3), indicating recent population growth while recovering from possible bottleneck. Values for Tajima's *D* were not significant in other sampling locations.

### Mitochondrial genetic population structure

**Figure 4:**
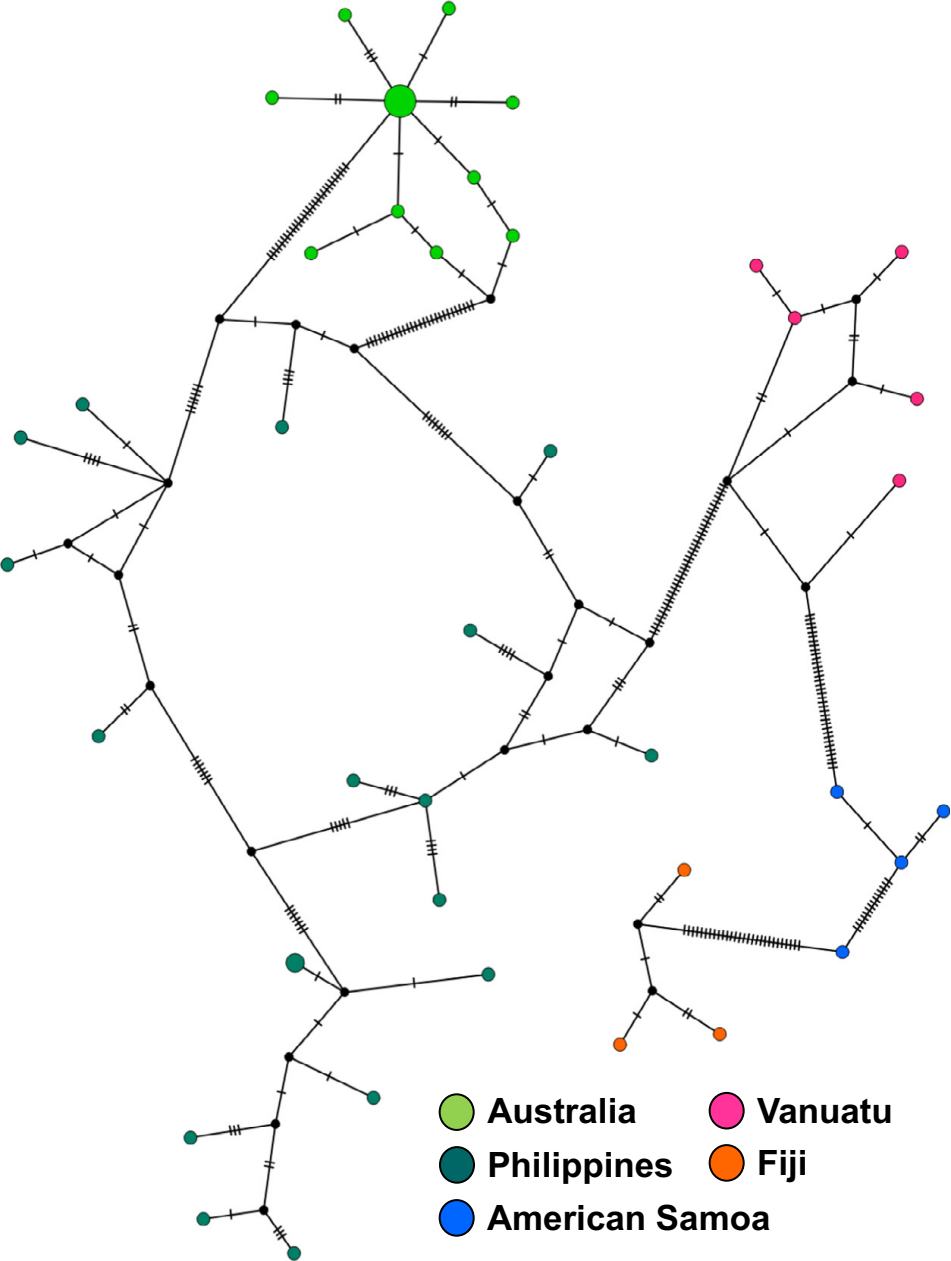
TCS haplotype networks of *Nautilus pompilius* from across the Indo‐Pacific that were collected for this study. Haplotype networks were constructed based on 1196 bp concatenated COI‐16S sequences (See Table S1 for sample information). Each circle represents a different haplotype, with its size proportional to the number of individuals found with that haplotype; black circles represent hypothetical ancestors; and dashes on branches indicate base pair differences. The sampling sites are indicated by different colors refer to the region in which haplotypes were found; we did not observe distinct haplotypes shared among regions.

TCS haplotype networks displayed for each locus revealed a strong association between haplotype variation and geographical distribution (Figs. 4 and S3). Haplotypes for concatenated *16S‐COI* sequences were not shared between geographical locations (Fig. 4). With the expanded *COI* data set of nautilus from across their range, haplotypes are heavily shared between Palau, Indonesia, eastern Australia, and the Philippines (Figure S3). No population was found to be completely isolated from other *N. pompilius* populations, including locations containing samples identified as other *Nautilus* species: *N. macromphalus*,* N. belauensis*,* N. stenomphalus*, and *N. repertus*.

## Discussion

There is an important conservation aspect to understanding and discerning the number of genetically distinct taxa in the extant Nautilidae: nautiluses are targeted for their shells and are sold in markets around the world as ornaments or jewelry (Sinclair et al. 2007, 2011; Dunstan et al. 2011a; De Angelis 2012). These fisheries are under no regulations to date, although a proposal to add the family Nautilidae to the CITES treaty under Appendix II protection has been proposed and will be reviewed in 2016. Increased information on the genetics and population biology of these species is vital in understanding the future of this living fossil and assessing the impact of the shell trade on populations across the Indo‐Pacific. Importantly, we need to know how rapidly (if at all) a local population driven to extinction by fishing might become repopulated from more distant localities. Lastly, due to the low fecundity and the unregulated fishing of these populations (Barord et al. 2014), local extinctions may be a real risk, as stressed by the CITES proposal.

Molecular data are paramount to understanding the present state of conservation crisis in nautilus. We have sought to resolve intergenus relationships within the genus *Nautilus*, as well as determine the number of extant species, as results from previous studies have been inconclusive (Sinclair et al. 2007; Bonacum et al. 2011; Sinclair et al. 2011). Our AMOVA results indicate that populations are relatively isolated from one another, with high variation across the range of *N. pompilius* being attributed to differences between populations, while the populations themselves are less diverse. This corresponds to the clustering of geographical regions within the TCS haplotype network, in which many geographical regions (particularly Fiji, American Samoa, New Caledonia, and Vanuatu) are relatively isolated with the highest numbers of base pair differences.

Our results add new samples to these important prior investigations; however, we differ from previous workers in our interpretation of current *Nautilus* taxonomy. The data presented here support that individual nautiluses identified as *N. belauensis*,* N. repertus*, and *N. stenomphalus* may be morphotypes or subspecies of *N. pompilius*, or individuals with interesting phenotypic plasticity, as the mitochondrial COI and 16S sequences from these nautiluses are indistinguishable from those of *N. pompilius* under rigorous phylogenetic analysis. Furthermore, original taxonomic descriptions of *N. belauensis*,* N. repertus*, and *N. stenomphalus* are based on a very small number of morphological characters. For example, a major morphological difference of the species *N. belauensis compared* to *N. pompilius* is a slightly different pattern of shell growth lines. Identifying solid taxonomic markers for distinguishing individuals from disparate populations as separate species is difficult because soft parts of these animals are considered to be identical, although we believe a rigorous taxonomic reassessment may illicit more informative morphological characters.

Further morphological and genetic experiments must be conducted to rigorously test this hypothesis. Identification of strong taxonomic characters from the viscera of extant nautilids would help to inform assessments of population‐ or species‐level differences at a morphological level. Microsatellites have recently been developed for *Nautilus* (Williams et al. 2015a, 2015b) but have not been employed across a wide geographical range. These powerful tools should be utilized to inform fisheries management and conservation efforts, as well as assess the validity of the currently recognized extant species.

## Conflict of Interest

None declared.

## Supporting information


**Figure S1.** Bayesian inference tree of *COI* sequences of *Nautilus pompilius* sequences from our study and sequences of all nonredundant *Nautilus* spp. sequences from GenBank (in bold); GenBank entries that had identical DNA sequences from the same collection location were not included in the analysis.

**Figure S2**. Bayesian inference tree of *16S* sequences of *Nautilus pompilius* from our study and sequences of *N. macromphalus* and *Allonautilus scrobiculatus* from GenBank.

**Figure S3.** Median‐joining network of COI haplotypes for *Nautilus* sp. from GenBank with 95% confidence.

**Table S1.** Descriptions and defining characters of *Nautilus* species examined in this study.

**Table S2.** Information for GenBank sequences from previous studies used in phylogenetic analysis including species identifications from previous studies, collection sites (country and specific location), Genbank accessions for *COI* or *16S*, and the publication or study that submitted the sequence.

**Table S3.** AMOVA of all *Nautilus* spp. COI sequences from GenBank.

**Table S4.** Population average pairwise differences for samples from this study, using concatenated 16S‐COI sequences.
