# Supplementary figures and images for: A revisited phylogeography of Nautilus pompilius
Source: Ecol Evol. 2016 Jun 21;6(14):4924–35. doi: 10.1002/ece3.2248 (PMC4979717; doi:10.1002/ece3.2248)

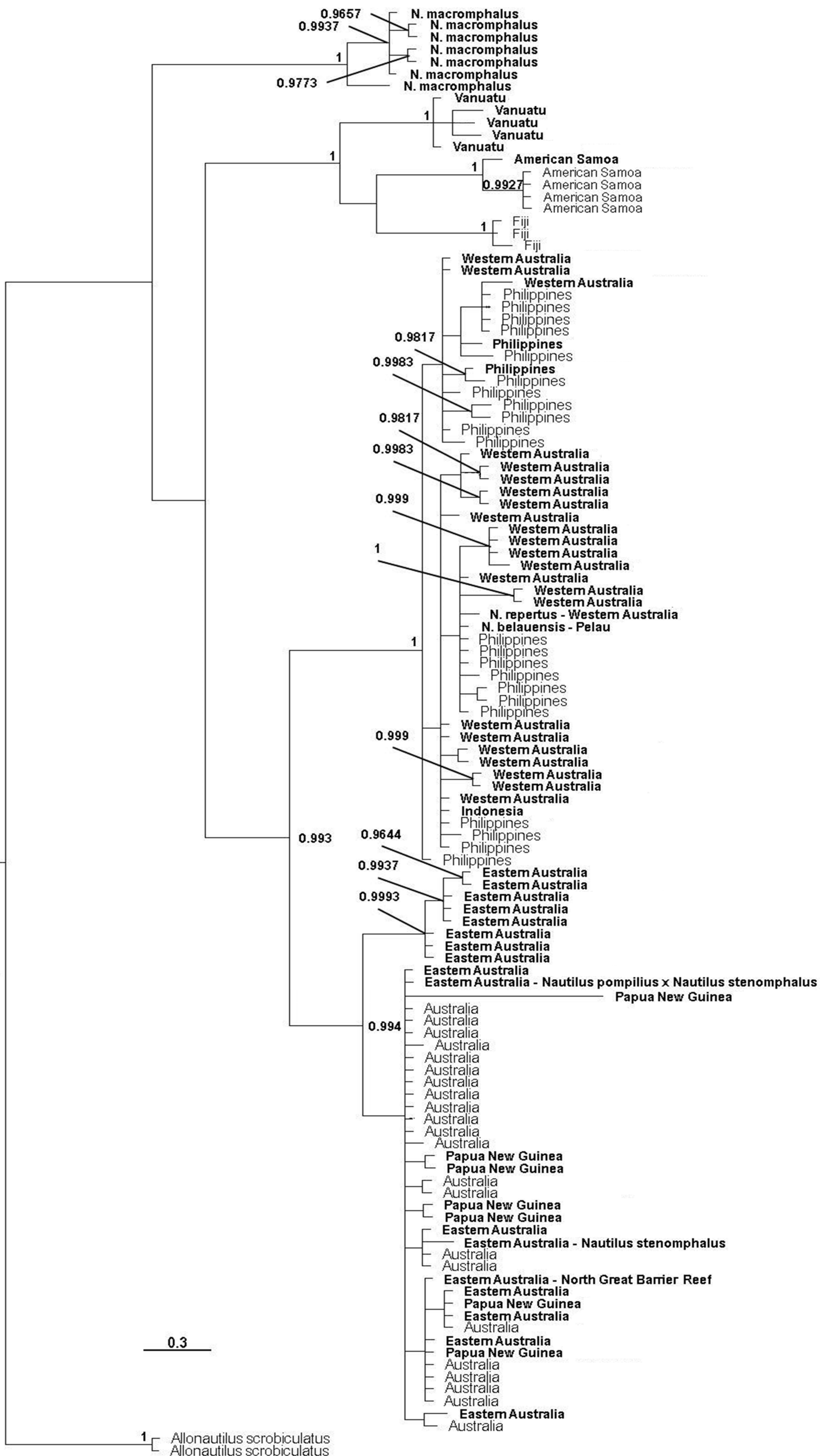

Supplement: Supplementary file 1 — Figure S1. Bayesian inference tree of COI sequences of Nautilus pompilius sequences from our study and sequences of all nonredundant Nautilus spp. sequences from GenBank (in bold); GenBank entries that had identical DNA sequences from the same collection location were not included in the analysis. [file ECE3-6-4924-s001.pdf]

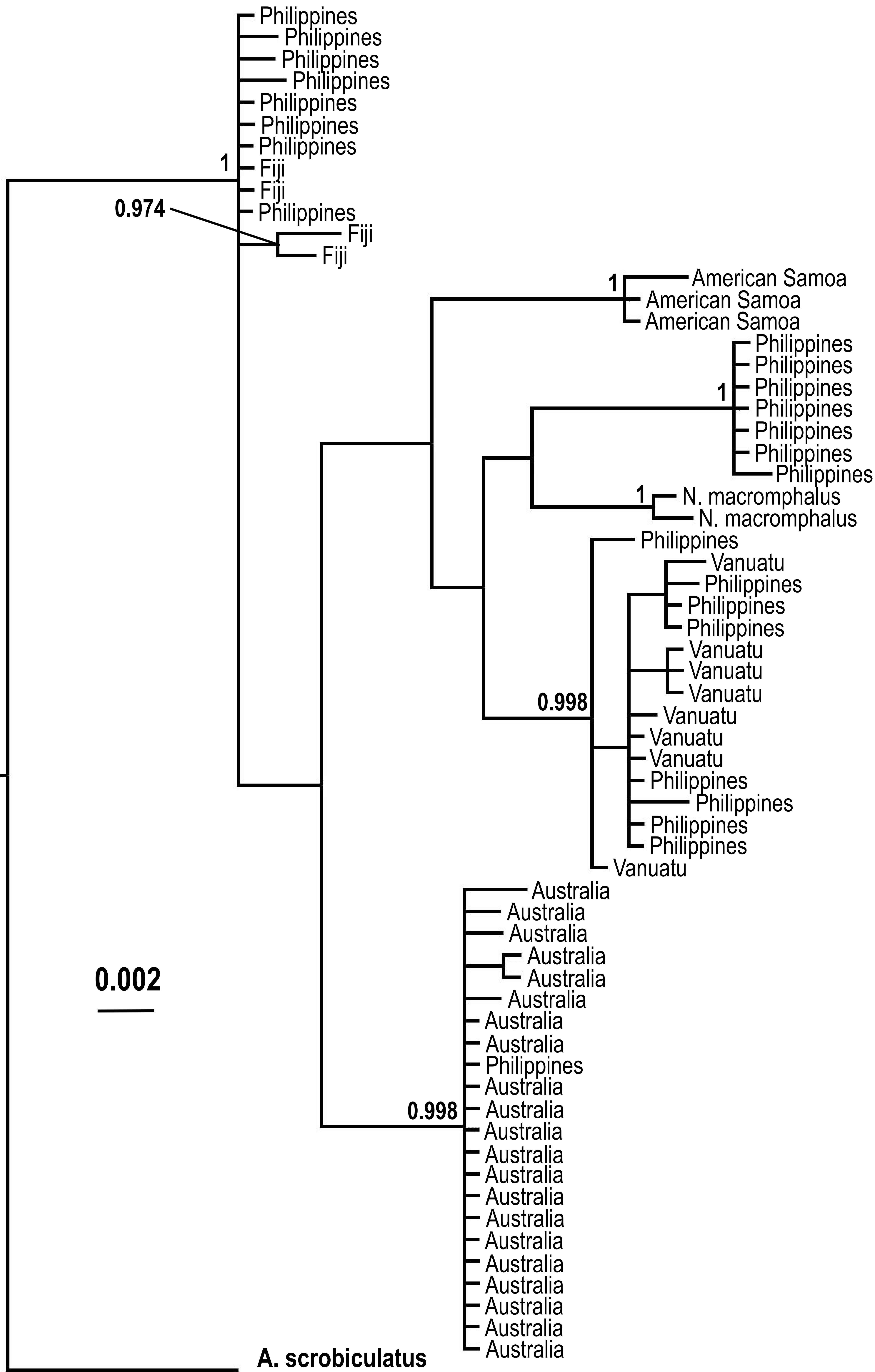

Supplement: Supplementary file 2 — Figure S2. Bayesian inference tree of 16S sequences of Nautilus pompilius from our study and sequences of N. macromphalus and Allonautilus scrobiculatus from GenBank. [file ECE3-6-4924-s002.pdf]

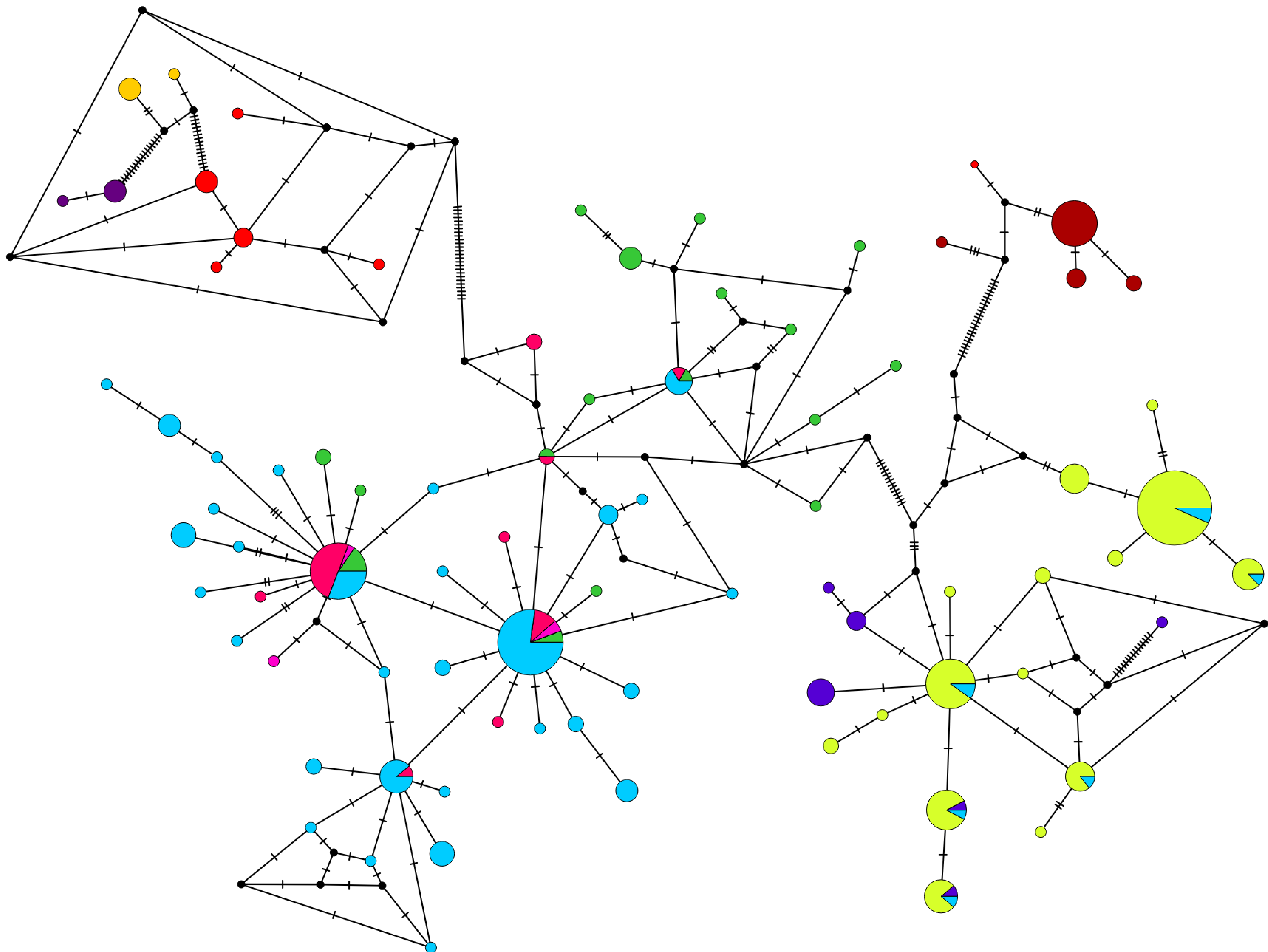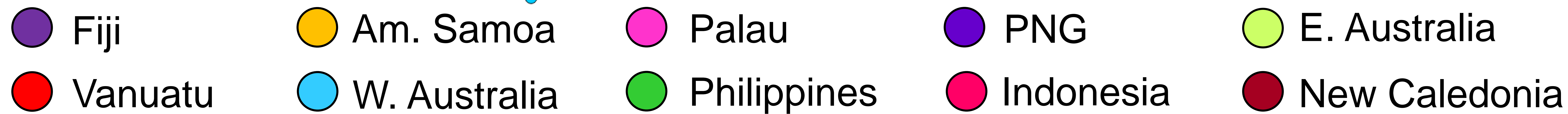

Supplement: Supplementary file 3 — Figure S3. Median‐joining network of COI haplotypes for Nautilus sp. from GenBank with 95% confidence. [file ECE3-6-4924-s003.pdf]
