# Supplementary material for: A revisited phylogeography of Nautilus pompilius
Source: Ecol Evol. 2016 Jun 21;6(14):4924–35. doi: 10.1002/ece3.2248 (PMC4979717; doi:10.1002/ece3.2248)
Supplement: Supplementary file 4 — Table S1. Descriptions and defining characters of Nautilus species examined in this study. [file ECE3-6-4924-s004.docx]

Table 1: Descriptions and defining characters of *Nautilus* species examined in this study

| Species | Type Locality | Mean weight | Shell Width Range | Mean Shell Width | | Defining characters |
| --- | --- | --- | --- | --- | --- | --- |
| *N. pompilius* Linnaeus, 1758 | Indonesia | 850 g | 115 – 199 mm | 170 mm | Type species of genus; size varies between populations; shell umbilicus 5% of shell diameter; shell color varies; hood texture varies; umbilical region less striped in southern populations | |
| *N. stenomphalus* Sowerby 1849 | Unknown | 605 g | 142 – 170 mm | 165 mm | Reduced shell coloration in umbilical region; typically lacks umbilical callous; textured hood with irregular papillae | |
| *N. macromphalus* Sowerby 1849 | New Caledonia? |  |  | 160 mm | Prominent, open umbilicus with inwardly sloping umbilical walls; shell umbilicus diameter 16% of shell diameter; similar shell coloration to *N. pompilius* | |
| *N. repertus* Linnaeus, 1758 | W. Australia |  | 184 – 243 mm | 220 mm | Shell form and umbilicus like *N. pompilius*; reduced coloration in umbilical region; lack of radial stripes in umbilical region | |
| *N. belauensis* Saunders, 1981 | Palau | 1300 g | 180 – 239 mm | 204 mm | Shell form and umbilicus like *N. pompilius*; shell has longitudinally crenulated ridges in reticulate pattern; broadly triangular rachidian radular tooth; size varies considerably within populations | |
