# Supplementary material for: A revisited phylogeography of Nautilus pompilius
Source: Ecol Evol. 2016 Jun 21;6(14):4924–35. doi: 10.1002/ece3.2248 (PMC4979717; doi:10.1002/ece3.2248)
Supplement: Supplementary file 5 — Table S2. Information for GenBank sequences from previous studies used in phylogenetic analysis including species identifications from previous studies, collection sites (country and specific location), Genbank accessions for COI or 16S, and the publication or study that submitted the sequence. [file ECE3-6-4924-s005.docx]

Supplementary Table 2: Information for GenBank sequences from previous studies used in phylogenetic analysis including species identifications from previous studies, collection sites (country and specific location), Genbank accessions for *COI* or *16S*, and the publication or study that submitted the sequence.

| **Species Identified** | **Collection Country** | **Collection Site** | **COI Accession** | **16S Accession** | **Citation** |
| --- | --- | --- | --- | --- | --- |
| Allonautilus scrobiculatus | Papua New Guinea | Little Ndrova Island | GQ280250 | GQ280184 | Bonacum et. al. 2011 |
| Allonautilus scrobiculatus | Papua New Guinea | Little Ndrova Island | GQ280251 | GQ280185 | Bonacum et. al. 2011 |
| Allonautilus scrobiculatus | Papua New Guinea | Little Ndrova Island | GQ280252 | GQ280186 | Bonacum et. al. 2011 |
| Nautilus macromphalus | New Caledonia | Ile des Pins | GQ280227 |  | Bonacum et. al. 2011 |
| Nautilus macromphalus | New Caledonia | Ile des Pins | GQ280228 |  | Bonacum et. al. 2011 |
| Nautilus macromphalus | New Caledonia | Ile des Pins | GQ280229 |  | Bonacum et. al. 2011 |
| Nautilus macromphalus | New Caledonia | Ile des Pins | GQ280230 |  | Bonacum et. al. 2011 |
| Nautilus macromphalus | New Caledonia | Ile des Pins | GQ280231 |  | Bonacum et. al. 2011 |
| Nautilus macromphalus | New Caledonia | Ile des Pins | GQ280232 | GQ280166 | Bonacum et. al. 2011 |
| Nautilus macromphalus | New Caledonia | Ile des Pins | GQ280233 | GQ280167 | Bonacum et. al. 2011 |
| Nautilus macromphalus | New Caledonia | Ile des Pins | GQ280234 | GQ280168 | Bonacum et. al. 2011 |
| Nautilus macromphalus | New Caledonia | Ile des Pins | GQ280235 | GQ280169 | Bonacum et. al. 2011 |
| Nautilus macromphalus | New Caledonia | Ile des Pins | GQ280236 | GQ280170 | Bonacum et. al. 2011 |
| Nautilus macromphalus | New Caledonia | Ile des Pins | GQ280237 | GQ280171 | Bonacum et. al. 2011 |
| Nautilus macromphalus | New Caledonia | Ile des Pins | GQ280238 | GQ280172 | Bonacum et. al. 2011 |
| Nautilus macromphalus | New Caledonia | Ile des Pins | GQ280239 | GQ280173 | Bonacum et. al. 2011 |
| Nautilus macromphalus | New Caledonia | Noumea | GQ280217 |  | Bonacum et. al. 2011 |
| Nautilus macromphalus | New Caledonia | Noumea | GQ280218 |  | Bonacum et. al. 2011 |
| Nautilus macromphalus | New Caledonia | Noumea | GQ280219 |  | Bonacum et. al. 2011 |
| Nautilus macromphalus | New Caledonia | Noumea | GQ280220 |  | Bonacum et. al. 2011 |
| Nautilus macromphalus | New Caledonia | Noumea | GQ280221 |  | Bonacum et. al. 2011 |
| Nautilus macromphalus | New Caledonia | Noumea | GQ280222 |  | Bonacum et. al. 2011 |
| Nautilus macromphalus | New Caledonia | Noumea | GQ280223 | GQ280157 | Bonacum et. al. 2011 |
| Nautilus macromphalus | New Caledonia | Noumea | GQ280224 | GQ280158 | Bonacum et. al. 2011 |
| Nautilus macromphalus | New Caledonia | Noumea | GQ280225 | GQ280159 | Bonacum et. al. 2011 |
| Nautilus macromphalus | New Caledonia | Noumea | GQ280226 | GQ280160 | Bonacum et. al. 2011 |
| Nautilus belauensis | Palau | - | GQ280187 | U11625.1 | Bonacum et. al. 2011 |
| Nautilus repertus | Australia: West | Rowley Shoals | GQ280188 | GQ280123 | Bonacum et. al. 2011 |
| Nautilus repertus | Australia: West | Rowley Shoals | GQ280189 | GQ280124 | Bonacum et. al. 2011 |
| Nautilus stenomphalus | Australia: East | Queensland: Carter Reef | GQ280196 | GQ280130 | Bonacum et. al. 2011 |
| Nautilus pompilius x stenomphalus | Australia: East | Queensland: Carter Reef | GQ280195 | U11603.1 | Bonacum et. al. 2011 |
| Nautilus pompilius | American Samoa | Pago Pago | GQ280214 |  | Bonacum et. al. 2011 |
| Nautilus pompilius | Australia: East | Queensland: Carter Reef | GQ280197 |  | Bonacum et. al. 2011 |
| Nautilus pompilius | Australia: East | Queensland: Carter Reef | GQ280198 |  | Bonacum et. al. 2011 |
| Nautilus pompilius | Australia: East | Queensland: Carter Reef | GQ280199 |  | Bonacum et. al. 2011 |
| Nautilus pompilius | Australia: East | Queensland: Carter Reef | GQ280200 |  | Bonacum et. al. 2011 |
| Nautilus pompilius | Fiji | Suva | GQ280215 |  | Bonacum et. al. 2011 |
| Nautilus pompilius | Fiji | Suva | GQ280216 |  | Bonacum et. al. 2011 |
| Nautilus pompilius | Indonesia | Ambon Strait | GQ280190 |  | Bonacum et. al. 2011 |
| Nautilus pompilius | Indonesia | Ambon Strait | GQ280191 |  | Bonacum et. al. 2011 |
| Nautilus pompilius | Papua New Guinea | Komuli Island | GQ280205 |  | Bonacum et. al. 2011 |
| Nautilus pompilius | Papua New Guinea | Little Ndrova Island | GQ280206 |  | Bonacum et. al. 2011 |
| Nautilus pompilius | Papua New Guinea | Little Ndrova Island | GQ280207 |  | Bonacum et. al. 2011 |
| Nautilus pompilius | Papua New Guinea | Little Ndrova Island | GQ280208 |  | Bonacum et. al. 2011 |
| Nautilus pompilius | Papua New Guinea | Little Ndrova Island | GQ280209 |  | Bonacum et. al. 2011 |
| Nautilus pompilius | Papua New Guinea | Little Ndrova Island | GQ280210 |  | Bonacum et. al. 2011 |
| Nautilus pompilius | Papua New Guinea | Little Ndrova Island | GQ280211 |  | Bonacum et. al. 2011 |
| Nautilus pompilius | Papua New Guinea | Little Ndrova Island | GQ280212 |  | Bonacum et. al. 2011 |
| Nautilus pompilius | Papua New Guinea | Little Ndrova Island | GQ280213 |  | Bonacum et. al. 2011 |
| Nautilus pompilius | Papua New Guinea | Lorengau | GQ280203 |  | Bonacum et. al. 2011 |
| Nautilus pompilius | Papua New Guinea | Lorengau | GQ280204 |  | Bonacum et. al. 2011 |
| Nautilus pompilius | Papua New Guinea | Port Moresby | GQ280201 |  | Bonacum et. al. 2011 |
| Nautilus pompilius | Papua New Guinea | Port Moresby | GQ280202 |  | Bonacum et. al. 2011 |
| Nautilus pompilius | Philippines | Pangalao Island | GQ280192 |  | Bonacum et. al. 2011 |
| Nautilus pompilius | Philippines | Balayan Bay | GQ280193 |  | Bonacum et. al. 2011 |
| Nautilus pompilius | Philippines | Balayan Bay | GQ280194 |  | Bonacum et. al. 2011 |
| Nautilus pompilius | Vanuatu | - | GQ280240 |  | Bonacum et. al. 2011 |
| Nautilus pompilius | Vanuatu | - | GQ280241 |  | Bonacum et. al. 2011 |
| Nautilus pompilius | Vanuatu | - | GQ280242 |  | Bonacum et. al. 2011 |
| Nautilus pompilius | Vanuatu | - | GQ280243 |  | Bonacum et. al. 2011 |
| Nautilus pompilius | Vanuatu | - | GQ280244 |  | Bonacum et. al. 2011 |
| Nautilus pompilius | Vanuatu | - | GQ280245 |  | Bonacum et. al. 2011 |
| Nautilus pompilius | Vanuatu | - | GQ280246 |  | Bonacum et. al. 2011 |
| Nautilus pompilius | Vanuatu | - | GQ280247 |  | Bonacum et. al. 2011 |
| Nautilus pompilius | Vanuatu | - | GQ280248 |  | Bonacum et. al. 2011 |
| Nautilus pompilius | Vanuatu | - | GQ280249 |  | Bonacum et. al. 2011 |
| Nautilus pompilius | Australia: West | Scott Reef | GQ387444 |  | Sinclair et. al. 2011 |
| Nautilus pompilius | Australia: West | Scott Reef | GQ387445 |  | Sinclair et. al. 2011 |
| Nautilus pompilius | Australia: West | Scott Reef | GQ387446 |  | Sinclair et. al. 2011 |
| Nautilus pompilius | Australia: West | Scott Reef | GQ387447 |  | Sinclair et. al. 2011 |
| Nautilus pompilius | Australia: West | Scott Reef | GQ387448 |  | Sinclair et. al. 2011 |
| Nautilus pompilius | Australia: West | Scott Reef | GQ387449 |  | Sinclair et. al. 2011 |
| Nautilus pompilius | Australia: West | Scott Reef | GQ387450 |  | Sinclair et. al. 2011 |
| Nautilus pompilius | Australia: West | Scott Reef | GQ387451 |  | Sinclair et. al. 2011 |
| Nautilus pompilius | Australia: West | Scott Reef | GQ387452 |  | Sinclair et. al. 2011 |
| Nautilus pompilius | Australia: West | Scott Reef | GQ387453 |  | Sinclair et. al. 2011 |
| Nautilus pompilius | Australia: West | Scott Reef | GQ387454 |  | Sinclair et. al. 2011 |
| Nautilus pompilius | Australia: West | Scott Reef | GQ387455 |  | Sinclair et. al. 2011 |
| Nautilus pompilius | Australia: West | Scott Reef | GQ387456 |  | Sinclair et. al. 2011 |
| Nautilus pompilius | Australia: West | Scott Reef | GQ387457 |  | Sinclair et. al. 2011 |
| Nautilus pompilius | Australia: West | Scott Reef | GQ387458 |  | Sinclair et. al. 2011 |
| Nautilus pompilius | Australia: West | Scott Reef | GQ387459 |  | Sinclair et. al. 2011 |
| Nautilus pompilius | Australia: West | Scott Reef | GQ387460 |  | Sinclair et. al. 2011 |
| Nautilus pompilius | Australia: West | Scott Reef | GQ387461 |  | Sinclair et. al. 2011 |
| Nautilus pompilius | Australia: West | Scott Reef | GQ387462 |  | Sinclair et. al. 2011 |
| Nautilus pompilius | Australia: West | Scott Reef | GQ387463 |  | Sinclair et. al. 2011 |
| Nautilus pompilius | Australia: West | Scott Reef | GQ387464 |  | Sinclair et. al. 2011 |
| Nautilus pompilius | Australia: West | Scott Reef | GQ387465 |  | Sinclair et. al. 2011 |
| Nautilus pompilius | Australia: West | Scott Reef | GQ387466 |  | Sinclair et. al. 2011 |
| Nautilus pompilius | Australia: West | Scott Reef | GQ387467 |  | Sinclair et. al. 2011 |
| Nautilus pompilius | Australia: West | Scott Reef | GQ387468 |  | Sinclair et. al. 2011 |
| Nautilus pompilius | Australia: West | Scott Reef | GQ387469 |  | Sinclair et. al. 2011 |
| Nautilus pompilius | Australia: West | Scott Reef | GQ387470 |  | Sinclair et. al. 2011 |
| Nautilus pompilius | Australia: West | Scott Reef | GQ387471 |  | Sinclair et. al. 2011 |
| Nautilus pompilius | Australia: West | Scott Reef | GQ387472 |  | Sinclair et. al. 2011 |
| Nautilus pompilius | Australia: West | Scott Reef | GQ387473 |  | Sinclair et. al. 2011 |
| Nautilus pompilius | Australia: West | Scott Reef | GQ387474 |  | Sinclair et. al. 2011 |
| Nautilus pompilius | Australia: West | Scott Reef | GQ387475 |  | Sinclair et. al. 2011 |
| Nautilus pompilius | Australia: West | Scott Reef | GQ387476 |  | Sinclair et. al. 2011 |
| Nautilus pompilius | Australia: West | Scott Reef | GQ387477 |  | Sinclair et. al. 2011 |
| Nautilus pompilius | Australia: West | Scott Reef | GQ387478 |  | Sinclair et. al. 2011 |
| Nautilus pompilius | Australia: West | Scott Reef | GQ387479 |  | Sinclair et. al. 2011 |
| Nautilus pompilius | Australia: West | Scott Reef | GQ387480 |  | Sinclair et. al. 2011 |
| Nautilus pompilius | Australia: West | Scott Reef | GQ387481 |  | Sinclair et. al. 2011 |
| Nautilus pompilius | Australia: East | North GBR | EF128174 |  | Sinclair et. al. 2011 |
| Nautilus pompilius | Australia: East | North GBR | EF128175 |  | Sinclair et. al. 2011 |
| Nautilus pompilius | Australia: East | North GBR | EF128176 |  | Sinclair et. al. 2011 |
| Nautilus pompilius | Australia: East | North GBR | EF128177 |  | Sinclair et. al. 2011 |
| Nautilus pompilius | Australia: East | North GBR | EF128178 |  | Sinclair et. al. 2011 |
| Nautilus pompilius | Australia: East | North GBR | EF128179 |  | Sinclair et. al. 2011 |
| Nautilus pompilius | Australia: East | North GBR | EF128180 |  | Sinclair et. al. 2011 |
| Nautilus pompilius | Australia: East | North GBR | EF128181 |  | Sinclair et. al. 2011 |
| Nautilus pompilius | Australia: East | North GBR: Mantis Reef | EF128182 |  | Sinclair et. al. 2011 |
| Nautilus pompilius | Australia: East | North GBR | EF128183 |  | Sinclair et. al. 2011 |
| Nautilus pompilius | Australia: East | Coral Sea: Osprey Reef | EF128184 |  | Sinclair et. al. 2011 |
| Nautilus pompilius | Australia: East | Coral Sea: Shark Reef | EF128185 |  | Sinclair et. al. 2011 |
| Nautilus pompilius | Australia: East | Coral Sea: Shark Reef | EF128186 |  | Sinclair et. al. 2011 |
| Nautilus pompilius | Australia: East | Coral Sea: Bougainville Reef | EF128187 |  | Sinclair et. al. 2011 |
| Nautilus pompilius | Australia: East | Coral Sea: Bougainville Reef | EF128188 |  | Sinclair et. al. 2011 |
| Nautilus pompilius | Australia: East | Coral Sea: Osprey Reef | EF128189 |  | Sinclair et. al. 2011 |
| Nautilus pompilius | Australia: East | Coral Sea: Shark Reef | EF128190 |  | Sinclair et. al. 2011 |
| Nautilus pompilius | Australia: East | Coral Sea: Shark Reef | EF128191 |  | Sinclair et. al. 2011 |
| Nautilus pompilius | Australia: East | Coral Sea: Shark Reef | EF128192 |  | Sinclair et. al. 2011 |
| Nautilus pompilius | Australia: East | Coral Sea: Shark Reef | EF128193 |  | Sinclair et. al. 2011 |
| Nautilus pompilius | Australia: East | Coral Sea: Shark Reef | EF128194 |  | Sinclair et. al. 2011 |
| Nautilus pompilius | Australia: East | Coral Sea: Shark Reef | EF128195 |  | Sinclair et. al. 2011 |
| Nautilus pompilius | Australia: East | Coral Sea: Shark Reef | EF128196 |  | Sinclair et. al. 2011 |
| Nautilus pompilius | Australia: East | Coral Sea: Osprey Reef | EF128197 |  | Sinclair et. al. 2011 |
| Nautilus pompilius | Australia: East | Coral Sea: Osprey Reef | EF128198 |  | Sinclair et. al. 2011 |
| Nautilus pompilius | Australia: East | Coral Sea: Osprey Reef | EF128199 |  | Sinclair et. al. 2011 |
| Nautilus pompilius | Australia: East | Coral Sea: Osprey Reef | EF128200 |  | Sinclair et. al. 2011 |
| Nautilus pompilius | Australia: East | Coral Sea: Osprey Reef | EF128201 |  | Sinclair et. al. 2011 |
| Nautilus pompilius | Australia: East | Coral Sea: Osprey Reef | EF128202 |  | Sinclair et. al. 2011 |
| Nautilus pompilius | Australia: East | Coral Sea: Osprey Reef | EF128203 |  | Sinclair et. al. 2011 |
| Nautilus pompilius | Australia: East | Coral Sea: Osprey Reef | EF128204 |  | Sinclair et. al. 2011 |
| Nautilus pompilius | Australia: East | Coral Sea: Osprey Reef | EF128205 |  | Sinclair et. al. 2011 |
| Nautilus pompilius | Australia: East | Coral Sea: Osprey Reef | EF128206 |  | Sinclair et. al. 2011 |
| Nautilus pompilius | Australia: East | Coral Sea: Osprey Reef | EF128207 |  | Sinclair et. al. 2011 |
| Nautilus pompilius | Australia: East | Coral Sea: Osprey Reef | EF128208 |  | Sinclair et. al. 2011 |
| Nautilus pompilius | Australia: East | Coral Sea: Osprey Reef | EF128209 |  | Sinclair et. al. 2011 |
| Nautilus pompilius | Australia: East | Coral Sea: Osprey Reef | EF128210 |  | Sinclair et. al. 2011 |
| Nautilus pompilius | Australia: East | Coral Sea: Shark Reef | EF128211 |  | Sinclair et. al. 2011 |
| Nautilus pompilius | Australia: East | Coral Sea: Osprey Reef | EF128212 |  | Sinclair et. al. 2011 |
| Nautilus pompilius | Australia: East | Coral Sea: Osprey Reef | EF128213 |  | Sinclair et. al. 2011 |
| Nautilus pompilius | Australia: East | Coral Sea: Osprey Reef | EF128214 |  | Sinclair et. al. 2011 |
| Nautilus pompilius | Australia: East | Coral Sea: Osprey Reef | EF128215 |  | Sinclair et. al. 2011 |
| Nautilus pompilius | Australia: East | Coral Sea: Osprey Reef | JN227630 |  | Williams et. al. 2012 |
| Nautilus pompilius | Australia: East | North GBR | JN227635 |  | Williams et. al. 2012 |
| Nautilus pompilius | Australia: West | Ashmore Reef | JN227639 |  | Williams et. al. 2012 |
| Nautilus pompilius | Australia: West | Ashmore Reef | JN227640 |  | Williams et. al. 2012 |
| Nautilus pompilius | Australia: West | Ashmore Reef | JN227641 |  | Williams et. al. 2012 |
| Nautilus pompilius | Australia: West | Ashmore Reef | JN227642 |  | Williams et. al. 2012 |
| Nautilus pompilius | Australia: West | Ashmore Reef | JN227643 |  | Williams et. al. 2012 |
| Nautilus pompilius | Australia: West | Ashmore Reef | JN227644 |  | Williams et. al. 2012 |
| Nautilus pompilius | Australia: West | Ashmore Reef | JN227645 |  | Williams et. al. 2012 |
| Nautilus pompilius | Australia: West | Ashmore Reef | JN227646 |  | Williams et. al. 2012 |
| Nautilus pompilius | Australia: West | Ashmore Reef | JN227647 |  | Williams et. al. 2012 |
| Nautilus pompilius | Australia: West | Ashmore Reef | JN227648 |  | Williams et. al. 2012 |
| Nautilus pompilius | Australia: West | Clerke Reef | JN227649 |  | Williams et. al. 2012 |
| Nautilus pompilius | Australia: West | Clerke Reef | JN227650 |  | Williams et. al. 2012 |
| Nautilus pompilius | Australia: West | Clerke Reef | JN227651 |  | Williams et. al. 2012 |
| Nautilus pompilius | Australia: West | Clerke Reef | JN227652 |  | Williams et. al. 2012 |
| Nautilus pompilius | Australia: West | Clerke Reef | JN227653 |  | Williams et. al. 2012 |
| Nautilus pompilius | Australia: West | Clerke Reef | JN227654 |  | Williams et. al. 2012 |
| Nautilus pompilius | Australia: West | Clerke Reef | JN227655 |  | Williams et. al. 2012 |
| Nautilus pompilius | Australia: West | Clerke Reef | JN227656 |  | Williams et. al. 2012 |
| Nautilus pompilius | Australia: West | Clerke Reef | JN227657 |  | Williams et. al. 2012 |
| Nautilus pompilius | Australia: West | Clerke Reef | JN227658 |  | Williams et. al. 2012 |
| Nautilus pompilius | Australia: West | Imperieuse Reef | JN227659 |  | Williams et. al. 2012 |
| Nautilus pompilius | Australia: West | Imperieuse Reef | JN227660 |  | Williams et. al. 2012 |
| Nautilus pompilius | Australia: West | Imperieuse Reef | JN227661 |  | Williams et. al. 2012 |
| Nautilus pompilius | Australia: West | Imperieuse Reef | JN227662 |  | Williams et. al. 2012 |
| Nautilus pompilius | Australia: West | Imperieuse Reef | JN227663 |  | Williams et. al. 2012 |
| Nautilus pompilius | Australia: West | Imperieuse Reef | JN227664 |  | Williams et. al. 2012 |
| Nautilus pompilius | Australia: West | Imperieuse Reef | JN227665 |  | Williams et. al. 2012 |
| Nautilus pompilius | Australia: West | Imperieuse Reef | JN227667 |  | Williams et. al. 2012 |
| Nautilus pompilius | Australia: West | Scott Reef | JQ890081 |  | Williams et. al. 2012 |
| Nautilus pompilius | Australia: West | Scott Reef | JQ890082 |  | Williams et. al. 2012 |
| Nautilus pompilius | Australia: West | Scott Reef | JQ890083 |  | Williams et. al. 2012 |
| Nautilus pompilius | Australia: West | Scott Reef | JQ890084 |  | Williams et. al. 2012 |
| Nautilus pompilius | Australia: West | Scott Reef | JQ890085 |  | Williams et. al. 2012 |
| Nautilus pompilius | Australia: West | Scott Reef | JQ890086 |  | Williams et. al. 2012 |
| Nautilus pompilius | Australia: West | Scott Reef | JQ890087 |  | Williams et. al. 2012 |
| Nautilus pompilius | Australia: West | Scott Reef | JQ890088 |  | Williams et. al. 2012 |
| Nautilus pompilius | Australia: West | Scott Reef | JQ890089 |  | Williams et. al. 2012 |
| Nautilus pompilius | Australia: West | Scott Reef | JQ890090 |  | Williams et. al. 2012 |
| Nautilus pompilius | Australia: East | Far North Great Barrier Reef | JQ862293 |  | Williams et. al. 2012 |
| Nautilus pompilius | Australia: East | Far North Great Barrier Reef | JQ862294 |  | Williams et. al. 2012 |
| Nautilus pompilius | Australia: East | Far North Great Barrier Reef | JQ862295 |  | Williams et. al. 2012 |
| Nautilus pompilius | Australia: East | Far North Great Barrier Reef | JQ862296 |  | Williams et. al. 2012 |
| Nautilus pompilius | Australia: East | Far North Great Barrier Reef | JQ862297 |  | Williams et. al. 2012 |
| Nautilus pompilius | Australia: East | Far North Great Barrier Reef | JQ862298 |  | Williams et. al. 2012 |
| Nautilus pompilius | Australia: East | Far North Great Barrier Reef | JQ862299 |  | Williams et. al. 2012 |
| Nautilus pompilius | Australia: East | Far North Great Barrier Reef | JQ862300 |  | Williams et. al. 2012 |
| Nautilus pompilius | Australia: East | Far North Great Barrier Reef | JQ862301 |  | Williams et. al. 2012 |
| Nautilus pompilius | Australia: East | Far North Great Barrier Reef | JQ862302 |  | Williams et. al. 2012 |
| Nautilus pompilius | Australia: East | Far North Great Barrier Reef | JQ862303 |  | Williams et. al. 2012 |
| Nautilus pompilius | Australia: East | Far North Great Barrier Reef | JQ862304 |  | Williams et. al. 2012 |
| Nautilus pompilius | Australia: East | Far North Great Barrier Reef | JQ862305 |  | Williams et. al. 2012 |
| Nautilus pompilius | Australia: East | Far North Great Barrier Reef | JQ862306 |  | Williams et. al. 2012 |
| Nautilus pompilius | Australia: East | Far North Great Barrier Reef | JQ862307 |  | Williams et. al. 2012 |
| Nautilus pompilius | Australia: East | Osprey Reef | JQ862308 |  | Williams et. al. 2012 |
| Nautilus pompilius | Australia: East | Osprey Reef | JQ862309 |  | Williams et. al. 2012 |
| Nautilus pompilius | Australia: East | Osprey Reef | JQ862310 |  | Williams et. al. 2012 |
| Nautilus pompilius | Australia: East | Osprey Reef | JQ862311 |  | Williams et. al. 2012 |
| Nautilus pompilius | Australia: East | Osprey Reef | JQ862312 |  | Williams et. al. 2012 |
| Nautilus pompilius | Australia: East | Osprey Reef | JQ862313 |  | Williams et. al. 2012 |
| Nautilus pompilius | Australia: East | Osprey Reef | JQ862314 |  | Williams et. al. 2012 |
| Nautilus pompilius | Australia: East | Osprey Reef | JQ862315 |  | Williams et. al. 2012 |
| Nautilus pompilius | Australia: East | Osprey Reef | JQ862316 |  | Williams et. al. 2012 |
| Nautilus pompilius | Australia: East | Osprey Reef | JQ862317 |  | Williams et. al. 2012 |
| Nautilus pompilius | Australia: East | Osprey Reef | JQ862318 |  | Williams et. al. 2012 |
| Nautilus pompilius | Australia: East | Osprey Reef | JQ862319 |  | Williams et. al. 2012 |
| Nautilus pompilius | Australia: East | Osprey Reef | JQ862320 |  | Williams et. al. 2012 |
| Nautilus pompilius | Australia: East | Osprey Reef | JQ862321 |  | Williams et. al. 2012 |
| Nautilus pompilius | Australia: West | Scott Reef | JQ862322 |  | Williams et. al. 2012 |
| Nautilus pompilius | Australia: West | Scott Reef | JQ862323 |  | Williams et. al. 2012 |
| Nautilus pompilius | Australia: West | Scott Reef | JQ862324 |  | Williams et. al. 2012 |
| Nautilus pompilius | Australia: West | Scott Reef | JQ862325 |  | Williams et. al. 2012 |
| Nautilus pompilius | Australia: East | Shark Reef | JQ862326 |  | Williams et. al. 2012 |
| Nautilus pompilius | Australia: East | Shark Reef | JQ862327 |  | Williams et. al. 2012 |
| Nautilus pompilius | Australia: East | Shark Reef | JQ862328 |  | Williams et. al. 2012 |
| Nautilus pompilius | Australia: East | Shark Reef | JQ862329 |  | Williams et. al. 2012 |
| Nautilus pompilius | Australia: East | Shark Reef | JQ862330 |  | Williams et. al. 2012 |
| Nautilus pompilius | Australia: East | Shark Reef | JQ862331 |  | Williams et. al. 2012 |
| Nautilus pompilius | Australia: East | Shark Reef | JQ862332 |  | Williams et. al. 2012 |
| Nautilus pompilius | Australia: East | Shark Reef | JQ862333 |  | Williams et. al. 2012 |
