# Supplementary material for: A revisited phylogeography of Nautilus pompilius
Source: Ecol Evol. 2016 Jun 21;6(14):4924–35. doi: 10.1002/ece3.2248 (PMC4979717; doi:10.1002/ece3.2248)
Supplement: Supplementary file 6 — Table S3. AMOVA of all Nautilus spp. COI sequences from GenBank. [file ECE3-6-4924-s006.docx]

**Supplementary Table 3:** AMOVA of all *Nautilus* spp. COI sequences from GenBank

| **Source of Variation** | d.f. | Sum of Squares | Variance Components | Percentage of Variation |
| --- | --- | --- | --- | --- |
| Among populations | 9 | 2392.9 | 9.7 Va | 85.52 |
| Within populations | 307 | 504.12 | 1.64 Vb | 14.48 |
| Total | 316 | 2867.01 | 11.34 |  |
| Fixation Index | F_ST_: 0.855 |  |  |  |
