# Supplementary material for: A revisited phylogeography of Nautilus pompilius
Source: Ecol Evol. 2016 Jun 21;6(14):4924–35. doi: 10.1002/ece3.2248 (PMC4979717; doi:10.1002/ece3.2248)
Supplement: Supplementary file 7 — Table S4. Population average pairwise differences for samples from this study, using concatenated 16S‐COI sequences. [file ECE3-6-4924-s007.docx]

Supplementary Table 4: Population average pairwise differences for samples from this study, using concatenated 16S-COI sequences.

Above diagonal : Average number of pairwise differences between populations (PiXY)

Diagonal elements: Average number of pairwise differences within population (PiX)

Below diagonal : Corrected average pairwise difference (PiXY-(PiX+PiY)/2)

|  | Australia | American Samoa | Vanuatu | Philippines | Fiji |
| --- | --- | --- | --- | --- | --- |
| Australia | 1.50476 | 52.95 | 47.46667 | 32.28889 | 48.73333 |
| American Samoa | 51.94762 | 0.5 | 25.85 | 51.13889 | 28.91667 |
| Vanuatu | 45.01429 | 23.9 | 3.4 | 46.32222 | 28.26667 |
| Philippines | 28.83063 | 48.18301 | 41.91634 | 5.41176 | 51.83333 |
| Fiji | 47.31429 | 28 | 25.9 | 48.46078 | 1.33333 |
